# A Common Representation of Spatial Features Drives Action and Perception: Grasping and Judging Object Features within Trials

**DOI:** 10.1371/journal.pone.0094744

**Published:** 2014-05-02

**Authors:** Jens H. Christiansen, Jeppe Christensen, Thor Grünbaum, Søren Kyllingsbæk

**Affiliations:** 1 Department of Psychology, University of Copenhagen, Copenhagen, Denmark; 2 Department of Media, Communication, and Cognition, University of Copenhagen, Copenhagen, Denmark; Emory University, United States of America

## Abstract

Spatial features of an object can be specified using two different response types: either by use of symbols or motorically by directly acting upon the object. Is this response dichotomy reflected in a dual representation of the visual world: one for perception and one for action? Previously, symbolic and motoric responses, specifying location, has been shown to rely on a common representation. What about more elaborate features such as length and orientation? Here we show that when motoric and symbolic responses are made within the same trial, the probability of making the same symbolic and motoric response is well above chance for both length and orientation. This suggests that motoric and symbolic responses to length and orientation are driven by a common representation. We also show that, for both response types, the spatial features of an object are processed independently. This finding of matching object-processing characteristics is also in agreement with the idea of a common representation driving both response types.

## Introduction

### Motoric responses and symbolic responses

The spatial features of a visual stimulus can be specified behaviorally using two distinct response types. One type can be referred to as a motoric response. This response type is made by directly acting motorically upon the stimulus. When making a motoric response there is a non-arbitrary relation between features of the stimulus and the correct response. As an example, when grasping a large object with thumb and index finger there is a larger distance between the fingers than when grasping a smaller object. When responding motorically, the participant's grip aperture indicates an estimation of size. Another type of response can be referred to as a symbolic response. In making a symbolic response, the participant is intentionally communicating a perceptual judgment using a symbol system. Using this type of response there can be, an arbitrary relation between the stimulus and the response. Pressing *key 1* on a keyboard when shown a large object and *key 2* when shown a small object is an example of a purely symbolic response to object size using an arbitrary mapping between response type and type of stimulus feature (arbitrary because the experimenter could just as well have decided to use the reverse mapping). When responding symbolically, the participant has typically been instructed to communicate his conscious perceptual estimation of specific stimulus features by the use of some symbol system.

Here we investigate the nature of the visual representations used to guide the two basic response types referred to as *symbolic responses* and *motoric responses*. More specifically, we investigate if these two basic response types are driven by common or separate visual representations of the spatial features of an object [1]–[5].

Answering this question is important for the ongoing debate concerning the degree of segregation of the neural processing streams underpinning symbolic and motoric responses. A prominent theory in this debate is the perception-action model developed by Milner and Goodale [1]. Seen through the optics of our current study the perception-action model exists in two versions: a version with strong segregation and a version with weak segregation. In the strong segregation version, the representations of spatial features of objects, which are driving motoric responses, are computed in isolation from the representations of spatial features of objects driving symbolic responses [1]–[3]. In the weak version a common representation of spatial features of objects is driving both motoric responses and symbolic responses [4], [5] (also, see commentary by Goodale and Milner in [6]). In this weak version of the perception-action model, the visual signals only separate for the purpose of *transforming* the representations of the spatial features of objects into formats suitable for making either a motoric or a symbolic response.

Answering the question of whether one common representation or two separate representations of the spatial features of an object drive the two response types will allow us to decide between the strong and the weak version of the perception-action model.

In the context of the perception-action model, motoric and symbolic responses are often referred to as actions and perceptual responses respectively (or estimates, reports or judgments [2], [7], [8]). An action can be both physical and mental. Therefore, the term *motoric response* is more precise since it relates only to a physical action. For this reason, we have chosen to use the term *motoric response*. Perceptual responses are responses based on perception. Perception is what happens when we become aware of a stimulus through sensory processing. Therefore, perception is more than awareness since it pertains to both sensory processing and awareness. By speaking of actions (such as motoric responses) vs. perceptual responses it is implicitly implied that the sensory processing behind awareness of a stimulus is different from the sensory processing behind an action (such as a motoric response). Since it is not known whether the sensory processes behind awareness of a stimulus are different from those behind a motoric response (it is indeed the very question we are trying to answer in the present paper), the use of the term *perceptual* is unfortunate.

In sum: the crucial distinction is not between “action” and “perception”, but rather between the participant making a direct motor response to the stimulus (e.g. grasping it or pointing to it) and the participant intentionally communicating his subjective estimation of some stimulus feature by making some kind of utterance (be it verbal or non-verbal, e.g. by pressing a button or manual gesture). To mark this important distinction in response types, we have chosen to use the terms “motoric responses” and “symbolic responses”.

### Previous findings

When testing whether representations of spatial features driving motoric and symbolic responses are common or separate, experimental paradigms have primarily been concerned with the question of whether select size-contrast illusions or specific types of brain damage differentially affect motoric and symbolic responses (for a recent review see [9]).

The rationale behind these experimental paradigms can be understood as follows: On the one hand, if the two response types are differentially affected by the illusion or the brain damage, then separate representations must have produced the responses. On the other hand, if the two response types are equally affected, then a common representation must have produced the responses.

Even though studies of size-contrast illusions or specific types of brain damage have been the most used paradigms, they have not been the most suited. Despite numerous experimental attempts to test whether representations are shared or separate, the arguments of the debate seem to have congealed around methodological issues (for examples see [10] vs. [11] or [12] vs. [13]).

Recognizing these difficulties in the debate Gegenfurtner and Franz [14] devised a different approach where they directly compared manual pointing (a motoric response) to judgments of location (a symbolic response). In their experiment, the task on each trial was to first point to a briefly presented Gaussian blob and then to judge whether the blob appeared to the left or right of two vertically aligned vertical lines placed above and below the blob. They found that agreement between pointing and judgments was well above chance. Based on this result, they concluded that a common representation of location drives both response types.

Exactly where this representation of location resides is not known but location is certainly represented as early as V1 in the retinotopic mapping of visual input from the eyes. What about representations of more elaborate features such as length and orientation, which are not directly represented in the retinotopic mapping? Do symbolic and motoric responses also share a common representation of these features?

According to Jeannerod's dual-channel hypothesis of grasping [15], the reach component of the grasp is independent of the parameters of the grip itself. This conclusion was based on the observation that unexpected changes in object size, during a grasping movement, led to corresponding changes in grip parameters without influencing the velocity profiles for the reach. If the parameters of reach (object location) and grip size (object size) are independently affected by the changes in object size then there might be differences between how a basic feature such as location and more elaborate features such as length and orientation drive symbolic and motoric responses.

Here we apply a paradigm and an analysis similar to the one used by Gegenfurtner and Franz [14] to the features length and orientation. The main result shows that the probability that the two response types are the same is well above chance for both features at a trial-by-trial basis. This result is fully in agreement with the theory of a common representation driving both symbolic and motoric responses to length and orientation. The result would, however, be very unlikely if symbolic and motoric response types were driven by separate representations.

## Methods

### The double response experiment

In the double response experiment participants made two types of responses during the same trial. First a grasping movement (motoric response) was made toward a briefly presented bar (the exposure duration was 47 ms) as soon as the bar was presented on a monitor and next the length and orientation of the same bar was reported using a keyboard (symbolic response).

The two response types used in the experiment are defined operationally as follows:

#### Motoric response

With minimal training/instruction participants were asked to move their index and thumb to the endpoints of the white bar as soon as the bar was presented (using about 1 sec of movement-time). The training was minimal because the act of making a motoric response in the present experiment was considered equal to the types of motoric responses participants make on a daily basis throughout their lives.

#### Symbolic response

Before the experiment proper began, participants went through an extensive learning process where each of four different lengths and each of five different orientations of the bars were associated to the numbers 1, 2, 3, and, 4 for length and 1, 2, 3, 4, and 5 for orientation. Upon presentation of a white bar a participant would then press e.g. 1 and 4 on a keyboard and thereby be specifying length 1 and orientation 4. The learning process was extensive because we wanted to train the participants until they got so fluent at making a symbolic responses that the novelty of the task did not interfere with their ability to correctly report the identity of the briefly presented bar.

### Analysis of the association of symbolic and motoric responses

When analyzing the observed alignment of symbolic and motoric responses, one must take into account that performance in both tasks depends on the visibility of the bar (i.e. floor or ceiling effects). One way to do this is to use a method suggested by Stone and Krauzlis [16] where expected chance alignments of the two response types are computed and compared to the observed number of aligned responses. Gegenfurtner and Franz [14] used this method to compare observed and expected (chance) alignment when pointing to a Gaussian blob and judging the location of the blob on the same trial, but they used a paradigm with binary response categories. We performed the analysis using a multinomial version where all responses were entered into the analysis.

The probability of obtaining the same motoric and symbolic response by chance 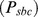
for a participant *x* when shown stimulus *n* is shown in Equation 1 where 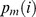 and 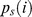 are the marginal probabilities of specifying the stimulus *n* as belonging to category *i* out of all possible categories *I* for motoric and symbolic responses, respectively. 
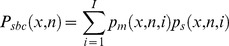
(1)


The number of occurrences where symbolic and motoric responses were the same (Probability-Same-Observed) was compared to the number of occurrences expected to have happened by chance (Probability-Same-by-Chance, 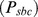). If the two response types are driven by independently computed lengths and orientations then the Probability-Same-Observed equals the Probability-Same-by-Chance. On the other hand, if a common representation drives the two response types, Probability-Same-Observed should be well above Probability-Same-by-Chance.

### Ethics Statement

All participants gave their informed consent in writing. The written consent was given in response to a document informing them, in detail, about their salary for participating, about what participating in the experiment consisted of and that, if they did not want to continue as participants, they could stop participating. The process is documented using an email-system and in prints of these emails. The Institutional Review Board at Department of Psychology, University of Copenhagen approved the study and the consent procedure and acknowledged that the study was conducted according to the declaration of Helsinki.

### Participants

We initially recruited seven participants (Group 1). Seven participants can rightfully be considered a small sample size. We therefore replicated the experiment with another group of seven participants (Group 2). The replication with Group 2 closely replicated the findings from Group 1 and thus confirmed the conclusions drawn from the initial results (see Results section). In the results section, the results from Group 1 are depicted in both figures and text. The results of Group 2 are reported in the text only. Group 1: Five female psychology students (age: 20–23 years. One left-handed) and two male authors (age 28 and 39 years. One left-handed). Group 2: Five female psychology students (age: 19–26 years) and two male psychology students (age 21 and 24 years). All were right-handed. In both groups all participants had normal or corrected-to-normal vision. All psychology students were paid for their participation and were naïve as to the purpose of the experiment. Group 1 included two authors. Being an author might have affected the results. The replications with seven naïve participants (Group 2) show that this justified concern was not a problem.

### Stimulus

The stimuli were always delivered on a 21-inch (19.8-inch visible) FD Trinitron CRT monitor (Model: Sony GDM-C520K), set at 85 Hz. The stimulus set used consisted of 20 different white bars of equal width (0.9 cm.). They had four lengths (6.6, 7.6, 8.6, and 9.6 cm.) and five orientations. There were both left-handed and right-handed participants. In order to equate the motoric response between these two groupings the bars were always oriented such that, for the right-hand group, the top end of the bar was inclined toward the left and for the left-handed group the top end of the bar was inclined towards the right. So for left-handed participants the orientations were 30°, 40°, 50°, 60°, and 70°. For the right-handed participants the orientations were 150°, 140°, 130°, 120°, and 110°. In pilot studies the size of the angular separation between the five different orientations was adjusted to be similar to the size between the four lengths in terms of difficulty when making a symbolic response (but, in terms of difficulty, we did not attempt to precisely equate a unit change in orientation to a unit change in length). Bars were presented at arms length for 47 ms (at 85 Hz a single frame lasted about 11.76 ms so four frames corresponded to about 47 ms). Pilot studies showed that this exposure duration was sufficient. Bars were presented on a black computer screen (one at a time on either the left or right side of a central fixation mark (a white cross) with the center of the bar located 7.5 cm. from the fixation mark). Because the length of the participants' arms differed, the precise visual degrees of the stimuli are not reported. A contrast adjustment procedure lowered the intensity of the bar until barely visible. When lowering the intensity of the bar, the aim was not to adjust the luminance to produce a certain proportion correct responses for length and orientation. The aim was to make sure that both features were sufficiently difficult to report correctly (not so hard that reporting them correctly would be at chance and not so easy that they would be reported correctly in every trial). The adjustment process is described in the procedure. The experiment took place in a semi-darkened room. E-prime (Psychology Software Tools, Pittsburgh, PA) was used for stimulus presentation and recording of the responses when participants were making symbolic responses.

### Apparatus

An Optotrak 3D investigator system (Northern Digital Inc., Waterloo, Ontario, Canada) was used to record movements and positions of the hand and fingers. At the beginning of a session, three infrared diodes were attached close to the tip of both the index finger and thumb. Participants then made slow and precise motoric responses to all 20 bars to each side of the fixation mark one at a time several times. The diodes were adjusted so that at least one pair of diodes (one on the thumb and one on the index finger) was registered by the Optotrak sensors when touching each of the 20 bars to both sides.

### Procedure

The seven participants in Group 1 completed two sessions. Each session consisted of five parts taking approximately two hours to complete:

#### Part 1

The participants were initially familiarized with the 20 bars presented one at a time to each side of the fixation mark in random order (this procedure was repeated three times in three blocks producing a total of 120 trials). Each bar was presented for as long as the participants needed while they fixated the fixation mark. Below the fixation mark the correct symbolic responses were displayed (1, 2, 3 or 4 for length and 1, 2, 3, 4 or 5 for orientation). Correct symbolic responses were always displayed with length as the first number and orientation the second. (luminance (CIE(1931)) of bars  = 1.13 cd/m^2^, luminance of the black background ≅0 cd/m^2^ (measured with the Cambridge Research Systems ColorCAL II).

#### Part 2

Next, all 20 bars were displayed one at a time to each side of the fixation in random order (this procedure was repeated three times in three blocks producing a total of 120 trials) (luminance (CIE(1931)) of bars  = 1.13 cd/m^2^, luminance of the black background ≅0 cd/m^2^). Presentation time was 47 ms and participants made a symbolic response after each presentation. Their answer was indicated beneath the fixation mark and afterwards the correct answer was indicated beneath their symbolic response. Correct symbolic responses were always displayed with length as the first number and orientation the second. This was also the order in which participants had to respond to the features of the bar.

#### Part 3

**Figure 1:**
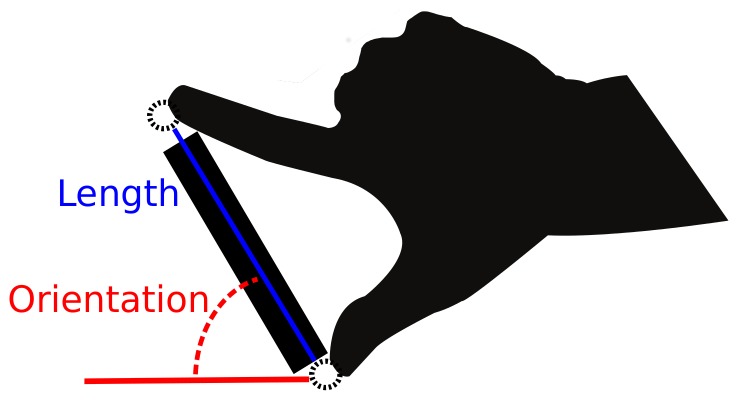
A motoric response to the length and orientation of a bar.

The calibration procedure. The orientation of the motoric response is defined as the angle between the horizontal plane and the virtual line between the index finger and thumb. Length is defined as the distance between the index and thumb finger (see Figure 1). Notice, though, that when attaching the infrared diodes close to the tip of both the index finger and thumb, the exact placement of the pair of diodes will vary between sessions and between participants. Therefore, in a single motoric response, the raw measure of distance between diodes and the angle between the horizontal plane and the virtual line between index finger and thumb cannot be directly related to a given length and orientation of a bar. That is, we can never know the true orientation category or length category to which a participant's raw motoric response corresponds. To be useful, the motoric responses need to be categorized using a classifier.

Before the motoric responses could be classified into the categories of the stimulus, a calibration procedure was performed. All 20 bars were presented once to both sides of the fixation mark in random order (this procedure was repeated two times in two blocks producing in total 80 trials). The bars were visible until the next trial was initiated and participants made a slow (about 1 sec.) and precise motoric response by placing their fingers on the endpoints of the bar. This way a given length and orientation was repeatedly measured 20 times. (luminance (CIE(1931)) of bars  = 1.13 cd/m^2^, luminance of the black background ≅0 cd/m^2^).

All subsequent motor responses of lengths and orientations were classified with a Naïve Bayes classifier [17], into four categories for length and five categories for orientation, using the prior obtained calibration data as training set (assuming equal prior probability among all response categories).

#### Part 4

Next, all 20 bars were presented once to both sides of the fixation mark in random order (this procedure was repeated four times in four blocks producing a total of 160 trials). Participants initiated presentation of the bar by pressing space. A one second delay was inserted between pressing space and presenting the bar. Again presentation time was 47 ms. In Part 4 stimulus luminance varied from trial to trial in an adaptive staircase manner.

If only one feature was incorrect the luminance was left unchanged. If both features were correct, the luminance was decreased and if both features were incorrect the luminance was increased. Increase and decrease were made according to the function:
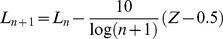
(2)


In the above equation 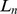 is the current luminance level in RGB values and 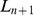
is the adjusted new luminance for next trial, *n* is trial number and *Z* takes the value 0 if both features were incorrect and 1 if both features were correct (modified from [18]).

Each time all 20 bars had been presented on both sides the function was restarted but with the last RGB setting inserted in the function. After each trial their answer was indicated beneath the fixation mark, but no feedback was given in terms of correct/incorrect.

#### Part 5

Next all 20 bars were presented once to both sides of the fixation mark in random order (this procedure was repeated four times in four blocks producing a total of 160 trials). The RGB level was fixed at the RGB level reached in Part 4. Part 5 constituted the measurement taken, in each trial, when making both a motoric and symbolic response. Each trial began when participants were ready and pressed space. After 1 second the white bar was presented for 47 ms at the RGB level reached in the adjustment in part 4. The RGB values had a mean luminance value of 0.02 cd/m^2^ and ranged between 0.01 and 0.06 cd/m^2^. As soon as the bar was presented participants moved their fingers from a resting position next to the keyboard to the endpoints of the bar, touching the screen and back again to the resting position. Immediately thereafter they made a symbolic response of length first and then orientation. The symbolic response was non-speeded. Each participant completed the above procedure twice in two sessions producing a total of 320 double response trials.

The luminance of the text, in all instructions given in the five different parts of a single session, was set at 1.13 cd/m^2^ to avoid adaptation that could interfere with the upcoming stimulus that was barely visible. The fixation mark always had the same luminance as the stimulus. For Group 2 Part 3 was performed before Part 1. Group 2 also completed two sessions. The median interval between the two sessions was eight days for Group 1 and four days for Group 2. According to a two-tailed t-test comparing the interval between session 1 and 2 for each participant in Group 1 to the interval between session 1 and 2 for each participant in Group 2 the interval was not significantly different for the two groups (t(12) 1.7447, P = 0.12).

## Results

### Categorization of motoric responses

**Figure 2:**
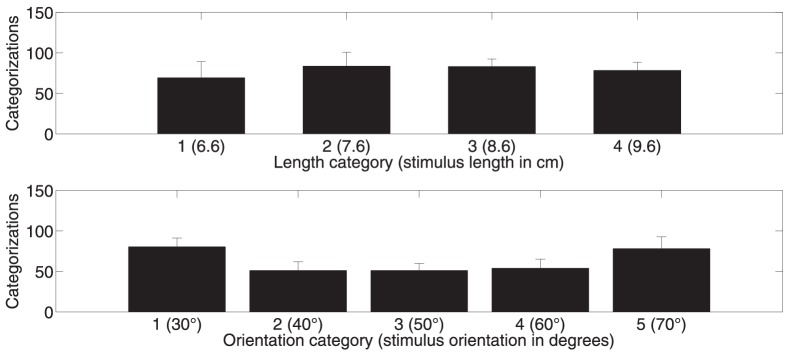
Categorization of motoric responses (for Group 1). Bars represent the average number of motoric responses to length and orientation across seven participants (a total of 2173 data points (2240 minus the 67 missing) categorized with a Naïve Bayes classifier. Top plot shows categorizations of length and bottom plot shows categorizations of orientation. Error-bars are SEM's across participants.

Figure 2 shows, for Group 1, the categorizations of motoric responses of lengths and orientations based on the Naïve Bayes classifier. Classification of orientation and length is performed after rejecting all trials ±3 standard deviations from the group mean. For Group 1, there were 28 missing symbolic length responses and 25 missing symbolic orientation responses (all 25 missing orientation responses coincided with the 28 missing length responses). Also, for Group 1, there were 58 missing motoric responses (there were no motoric responses where only one feature was missing). 9 of the missing symbolic responses did not coincide with the 58 missing motoric responses. Therefore, there were 67 missing double responses out of 2240. For Group 2, there were 15 missing symbolic length responses and 6 missing symbolic orientation responses (all 6 missing orientation responses coincided with the 15 missing length responses). Also, for Group 2, there were 28 missing motoric responses (there were no motoric responses where only one feature was missing). One of the missing symbolic responses did not coincide with the 28 missing motoric responses. Therefore, there were 29 missing double responses out of 2240.

Given that we can never know the true orientation category or length category of a participants motoric response (see Procedure: Part 3), we cannot asses the classifier performance in the classical sense that involve supervised testing of how well it performs for each category. However, we can look at how well the participants perform the task given the classifier at hand. If we assume the classifier works no better than chance (randomly chooses a length and orientation category for each response), the participant performance would not exceed 25% for length and 20% for orientation. The proportion correct for lengths were: Group 1(0.50, 0.40, 0.39, 0.56), Group 2 (0.64, 0.34, 0.34, 0.66) and for orientations: Group 1 (0.85, 0.53, 0.60, 0.54, 0.84), Group 2 (0.84, 0.51, 0.61, 0.65, 0.85). To test if the proportions were above chance, we performed a binominal test for each length and orientation category for each participant. We then transformed the derived p-values from these tests into χ^2^-square values to test the effect across participants [19]. All of these tests were highly significant, thus, the classifier is performing well above chance level.

To further evaluate the classifier's performance, we analyzed if participants' proportion correct was significantly different between motoric and symbolic responses. We evaluated each category with a paired t-test statistic for both groups (N = 7 in each group), H0: Performance(motoric)  =  Performance(symbolic). For length: Group 1 (p = 0.81, 0.26, 0.08, 0.06), Group 2 (p = 0.2, 0.16, 0.007*, 0.16) and for orientation: Group 1 (p = 0.67, 0.9, 0.69, 0.26, 0.35), Group 2 (p = 0.11, 1, 0.7, 0.1, 0.38) (two-tailed, α = 0.05). Only one condition show a significantly different performance between motoric and symbolic response evaluated with a parametric test. In Table 1, overall proportion correct length and orientation are shown for Group 1 and Group 2.

**Table 1:** Mean proportion correct and standard deviations () for length and orientation across participants in a given group for a given response type.

Proportion correct (%)			
Response type		Group 1 (N = 7)	Group 2 (N = 7)
**Length**	*Motoric*	46.2 (±7.9)	49.3 (±6.9)
	*Symbolic*	47.3 (±7.4)	50.2 (±6.4)
**Orientation**	*Motoric*	66.8 (±10.5)	69.3 (±5.3)
	*Symbolic*	66.5 (±4.1)	63.6 (±7.4)

In order to examine if there were any differences between the overall proportion correct motoric and symbolic responses depicted in Table 1 a two-way repeated measures ANOVA was performed on the proportion correct motoric and symbolic responses for overall length and overall orientation (response type(symbolic vs. motoric) x (feature type(length vs. orientation)). For Group 1, there was a main effect of feature type (F(1,6) = 134.252, p<0.0001, ηp2 = 0.957); no main effect of response type (F(1,6) = 0.041, p = 0.846); and no interaction F(1,6) = 0.115, p = 0.746. For Group 2, there was a main effect of feature type (F(1,6) = 39.761, p = 0.001, ηp2 = 0.869); no main effect of response type (F(1,6) = 0.734, p = 0.424); and no interaction F(1,6) = 3.389, p = 0.115. Collapsing Group 1 and 2 produced the same result with a main effect of feature type (F(1,13) = 134.093, p<0.0001, ηp2 = 0.912); no main effect of response type (F(1,13) = 0.336, p = 0.527); and no interaction F(1,13) = 2.055, p = 0.175. These analyses tell us that the classifier introduces no additional error to the classification of the motoric responses than the one already there.

The Naïve Bayes classifier was chosen because it assumes independency among categories and imposes a uniform prior – hence on its own, it introduces no bias. However, this does not mean that there is no response bias in the motoric responses. In fact, there is response bias in both motoric and symbolic responses for both length and orientation. This can be shown with a χ^2^-square test evaluating the extent to which the distribution of motoric or symbolic responses to length or orientation differ from a completely unbiased distribution. In a completely unbiased distribution, the expected number of responses for each length category is (total number of length responses)/(4) and (total number of orientation responses)/(5) for each orientation category. We performed the χ^2^-square test on each participant, summed the χ^2^-values across participants and from this value determined if distributions of motoric or symbolic responses to length or orientation differed significantly from the unbiased expected distributions. In all instances, they did. (Group 1 (motoric length χ^2^ (21) = 148, p<0.0001); motoric orientation χ^2^ (28) = 163, p<0.0001), symbolic length χ^2^ (21) = 475, p<0.0001), symbolic orientation χ^2^ (28) = 138, p<0.0001)) and (Group 2 (motoric length χ^2^ (21) = 114, p<0.0001); motoric orientation χ^2^ (28) = 97, p<0.0001), symbolic length χ^2^ (21) = 245, p<0.0001), symbolic orientation χ^2^ (28) = 121, p<0.0001)).

### Response-time

On average, participants in Group 1 used 1.289 seconds (*SD* = 0.16) from presentation of the bar and until touching the screen. In the replication performed using Group 2 the reaction times had an average of 1.275 seconds (*SD* = 0.23). A two tailed independent samples t-test showed that the response-times of the groups were not significantly different (*t*(12) = 0.135, *p* = 0.89). Within the context of the current experimental design, the response-times can be considered adequate: they are neither to fast nor to slow. On the one hand, when participants were instructed on how to perform the motoric response, they were told to move their index and thumb to the endpoints of the white bar as soon as the bar was presented using about 1 sec of movement-time. We wanted to avoid that participants viewed the motoric response as a fast-paced reaction-time task because the symbolic response, to which it was compared, was unspeeded. On the other hand, we did not want the motoric response to be performed in “slow motion” since this would have introduced a delay of several seconds between removal of the white bar and the final position of the fingers on the screen of the monitor. For reasons discussed later in the section *A note on the use of brief exposure durations* , such a delay would have been problematic.

### Above chance agreement between symbolic and motoric responses

**Figure 3:**
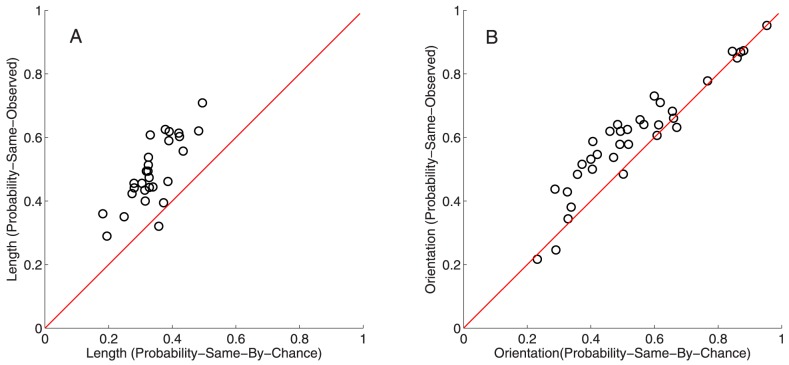
Comparison of Probability-Same-by-Chance and Probability-Same-Observed for length (A) and orientation (B) (for Group 1). In Panel A the black circles show the probability of obtaining the same motoric and symbolic response by chance (x-axis) compared to the observed probability (y-axis). Data-points falling above the red y = x line indicate instances where symbolic and motoric responses are in agreement above chance. Panel B shows the same for orientation.

In Figures 3a and 3b, the Probability-Same-Observed is plotted on the y-axis and the Probability-Same-by-Chance (derived from equation 1) is plotted on the x-axis (for Group 1). Figure 3a contains 28 data-points 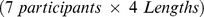 and each data-point consists of 80 trials (2 sessions of 160 trials each/4 lengths). Figure 3b has 35 data-points 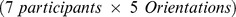 and each data-point consists of 64 trials (2 sessions of 160 trials each/5 orientations). If data-points fall on the diagonal y = x line, symbolic and motoric responses are no more in agreement than what can be expected by chance. Any point falling above this line suggests that symbolic and motoric responses are more in agreement than what can be expected by chance. For length almost all data-points lie well above chance and for orientation the majority lies above chance.

We compared Probability-Same-by-Chance and Probability-Same-Observed values for both length and orientation using the Wilcoxon signed rank test (for both Group 1 and Group 2). For Group 1 both tests showed that mean Probability-Same-Observed was significantly above mean Probability-Same-by-Chance (Length: Probability-Same-by-Chance (*mean  = 0.3444*) and Probability-Same-Observed (*mean  = 0.4821*) W = 404, p<0.001; Orientation: Probability-Same-by-Chance (*mean  = 0.5391*) and Probability-Same-Observed (*mean  = 0.5997*) W = 561, p<0.001). For Group 2 both tests showed a replication of the findings in Group 1. For Group 2 mean Probability-Same-Observed was significantly above mean Probability-Same-by-Chance (Length: Probability-Same-by-Chance (*mean  = 0.3732*) and Probability-Same-Observed (*mean  = 0.5165*) W = 406, p<0.001; Orientation: Probability-Same-by-Chance (*mean  = 0.5253*) and Probability-Same-Observed (*mean  = 0.5765*) W = 520, p<0.001).

### On the distribution of proportion correct and number of responses along the four lengths and the five orientations: comparing symbolic and motoric responses

To examine our data further, we tested whether motoric and symbolic responses showed different patterns of proportion correct or categorization biases. We used a χ^2^-test to compare the distributions of proportion correct symbolic and motoric responses along the four length categories or along the five orientation categories. We performed the χ^2^-test for each participant and summed the χ^2^-values to test the effect across participants (within Group 1 or 2). (Group 1 (length χ^2^ (21) = 101, p<0.0001); orientation χ^2^ (28) = 31, p<0.31), and (Group 2 (length χ^2^ (21) = 114, p<0.0001); orientation χ^2^ (28) = 44, p<0.023). We also use a χ^2^-test to compare the distributions of the number of motoric and symbolic responses along either the four lengths or the five orientations. Again we performed the χ^2^-test for each participant and sum the χ^2^-values to test the effect across participant (within Group 1 or 2). (Group 1 (length χ^2^ (21) = 355, p<0.0001); orientation χ^2^ (28) = 107, p<0.0001), and (Group 2 (length χ^2^ (21) = 378, p<0.0001); orientation χ^2^ (28) = 105, p<0.0001).

Thus, three out of four distributions of proportion correct responses were significantly different between response types and all the distributions of the number of responses (along the four lengths and the five orientations) were significantly different. Can these differences by themselves tell us whether or not a common representation drives the two response types? As noted in the introduction, there are two versions of the perception-action model by Milner and Goodale: A weak segregation version and a strong segregation version. Evidence showing differences in error patterns and categorization biases for motoric and symbolic responses might, on the face of it, be taken as evidence for the strong segregation version, but the very same differences in error patterns and categorization biases are just as likely to occur under the weak segregation version. The reason is that even in the weak segregation version the visual signals must at some point travel separate routes in order to transform the common representation of the spatial features of objects into a format suitable for making either a motoric or a symbolic response. According to the weak segregation version, information from a common representation is therefore fed into two segregated streams. Within these streams they must be transformed into motoric responses (dorsal) and symbolic responses (ventral). Independent sources of noise can affect the separate transformations and the two segregated transformations will sometimes be erroneous and can have different biases. This means that there can be differences between distributions of motoric and symbolic responses (both in number and in accuracy) even when the responses originate in a common representation.

To decide between the two versions of the perception-action model, we must instead look beyond the noise and the response bias that will be a part of the transformation of the representation of the bar into each of the two response types, also according to the weak segregation model. This is what we have done in Figure 3. If the strong segregation version of the perception-action model was correct and each of the two response types were based on independently computed representations of length (or orientation), then the Probability-Same-by-Chance should be equal to the Probability-Same-Observed. This is not what we find. Instead we find that the Probability-Same-Observed, for a given participant when shown a particular category of a feature, is well above the Probability-Same-by-Chance.

### Independent feature processing during both motoric and symbolic responses

If motoric and symbolic responses are driven by a common representation then basic characteristics of the representation should be reflected in both types of responses.

One example of this is the finding that the visual representation used to guide motoric responses in the absence of visual feedback shows the same decay characteristics (exponential decay) as typically found when asking participants to perform symbolic responses [20].

Another way to analyze the internal structure of a representation of an object is in terms of whether or not the features composing the object are processed dependently or independently. That two features of one object are processed independently should be understood as follows: In any given trial, the probability of making a correct response to one of the features will depend on the marginal probability of making a correct response to that feature only. It will not depend on whether the response to the other feature was correct. If it did depend on whether the other feature was specified correctly, then the two features were processed *dependently* of each other. Even though the specific pairing of length and orientation for a symbolic response has not, to our knowledge, been analyzed in terms of independence, many other feature-pairs have been examined. It is an established hallmark of symbolic responses to features of an object that they are processed independently (e.g. [21]). We therefore expect that, in terms of correctness, symbolic responses to length will be independent from symbolic responses to orientation.

In order to answer the question of independence, all data from Group 1 was converted into correct/incorrect answers and these answers were entered into a multinomial test of independence [21]. Seven participants had completed a total of 14 blocks (of 160 trials) for both symbolic and motoric responses. Therefore we conducted 28 multinomial tests of independence. A non-significant p-value (above alpha  = 0.05) was used as a measure indicating independence. In all 28 instances, the multinomial test of independence indicated independent processing of the two features. The results from Group 2 were also analyzed using the multinomial test of independence. This also produced 28 non-significant p-values. Thus, 56 times out of 56, the multinomial tests of independence failed to find a significant dependence within a given response type,between correct/incorrect judgments of length and orientation of a stimulus.

The results of the multinomial test can be illustrated by comparing the probability of getting both length and orientation correct in a given trial to the observed probability.

**Figure 4:**
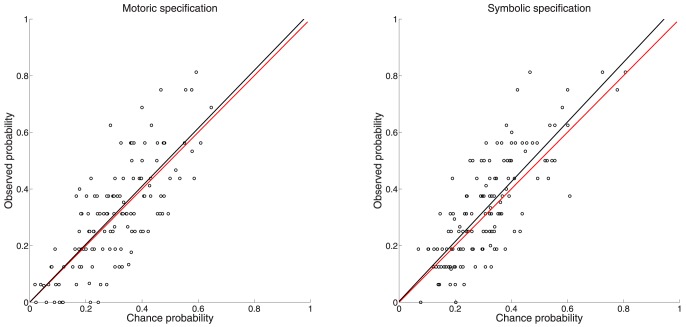
Comparing the chance probability and the observed probability of getting both length and orientation correct within response types (for Group 1). In each panel the y-value of a data-point is the proportion of trials, for a given participant responding to a given combination of length and orientation, where both length and orientation were correct. Therefore, in total, there are 140 data-points in each panel (seven participants performing each of the 20 combinations of length and orientation). Each data-point is based on 16 trials. The x-value of a data-point is derived from a model of independence. It is calculated by multiplying the marginal probability of getting a specific length correct (across all orientations) with the marginal probability of getting a specific orientation correct (across all lengths). The specific length and orientation is chosen so that it corresponds to the combination of length and orientation used to derive the y-value. The red line is y = x and the grey line is the best linear fit. The lines show how closely matched the observed probability is to the chance probability. For motoric responses in Group 1 the slope of the grey line was 1.023 (and intersected the y-axis at 0.000). For symbolic responses in Group 1 the slope of the grey line was 1.055 (and intersected the y-axis at 0.0032). For motoric responses in Group 2 the slope of the grey line was 1.011 (and intersected the y-axis at 0.01). For symbolic responses in Group 2 the slope of the grey line was 1.096 (and intersected the y-axis at 0.0252).

In Figure 4, this chance probability of getting both length and orientation correct is compared to the observed probability of getting both correct and depicted separately for motoric and symbolic responses (for Group 1). The observed probability is well predicted by the chance probability. Therefore, length and orientation are specified independently in both the motoric and the symbolic responses. This finding was replicated using Group 2.

### Standard deviation of motoric responses to briefly presented 2D objects does not follow Weber's law

As mentioned in the introduction, the perception-action model is a leading theory in the ongoing debate concerning the degree of segregation of the neural processing streams underpinning symbolic and motoric responses. In the perception-action model, visual input separate into two streams at a low level in the visual system (in V1+; the + is from [22] and we take it to mean “early visual cortex not otherwise specified”). One stream flows along a dorsal route where visual information is transformed and represented in a format supposedly suitable for connecting with the motor system. That is, suitable for performing actions on objects based on vision which according to the perception-action model necessitates a metric in egocentric coordinates. Another stream flows along a ventral route where visual information is transformed and represented in a format suitable for connecting with memory systems and conscious awareness. In order to do this, this stream is using a metric with more emphasis on objects relative to other objects [1].

**Figure 5:**
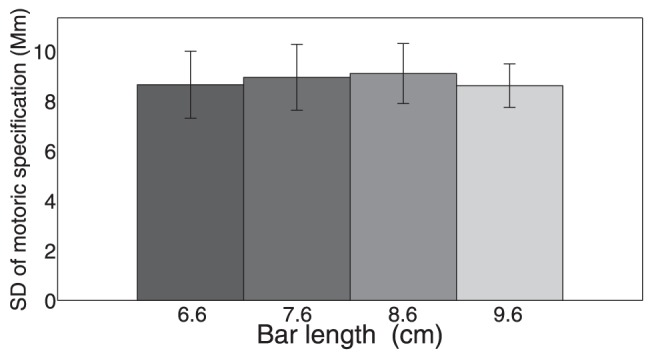
The standard deviations of motoric responses to length for Group 1. The histogram above shows the standard deviations of motoric responses, from the double response experiment, at four different lengths. Each bar is the mean standard deviation calculated pr. participant (N = 7). Each bar is based on approximately 560 motoric responses. Error-bars are SEM's across participants. The y-axis is in mm. A straight line fitted through the four mean standard deviations had a slope of 0.0029 mm (and intersected the y-axis at 8.8 mm). For Group 2 the best fitting straight line even had a negative slope of −0.3 mm (and intersected the y-axis at 13 mm). The analysis of standard deviations replicates earlier findings using 3D objects [23 see figure 1A] by showing that standard deviations of motoric responses to length, as length is increased, does not follow Weber's law. In fact, a slope of 0.0029 mm means that the averages of the standard deviations are almost identical across the different lengths used. The findings in [23], showing that the standard deviations of grasping 3D objects does not follow Weber's law, was based on a condition where 11 participants grasped 3D objects with lengths of 2, 3, 4, 5, 6, and 7 cm (note that in the study it is erroneously reported that 13 participants completed the condition). In [23] a one-way ANOVA was conducted (six lengths and 11 participants) and reported as F(5,60)<1. Based on the non-significant result of the one-way ANOVA the authors in [23] concluded that the standard deviations for grasping was not affected by object size. In our study a total of 14 participants (Group 1+Group 2) grasped 2D objects with lengths of equal increments (6.6, 7.6, 8.6, 9.6 cm). Having a similar number of participants and lengths of equal increments we also conducted a one-way ANOVA with a result similar to what was reported in [23] [F(3,52) = 0.105,p = 0.957]. The observed violation of Weber's law, during grasping, has been proposed as a marker for dorsal stream processing [24], [25] Thus, according to this proposal, the dorsal stream drove the motoric responses in the present experiment.

It has been argued that the dorsal stream, thought to drive motoric responses, is only properly activated when grasping real 3D objects that are visible until the grasp is completed (see the sections in the discussion titled: *A note on the use of brief exposure durations* and *a note on the use of 2D stimuli* for further discussion of these topics). According to these arguments the brief exposure durations and 2D stimuli we used might have failed to activate the dorsal stream. To investigate if the dorsal stream is fully activated by the briefly presented 2D stimuli used in the present work we analyzed the variability of the motoric responses to length as lengths were increased (lengths were 6.6, 7.6, 8.6, and 9.6 cm.). The analysis was inspired by the finding that grasp precision of 3D objects does not follow Weber's law [23]. This has been proposed as a marker for dorsal stream processing [24], [25] as opposed to symbolic responses to length which do follow Weber's law [23], [26] and are thought to be driven by ventral stream processing. If the motoric responses in the present experiment also follows Weber's law then it can be argued that ventral stream processing are driving the motoric responses to 2D objects. If so a well-above-chance agreement of the two response types, in the present experiment, is expected since they might both be driven by the same representation of length situated in the ventral stream [1]–[5], [24]. According to Weber's law, the *just-noticeable difference* between two stimuli increases as the magnitude of the stimuli increases (measured using symbolic responses!). Of course, when the *just-noticeable difference* between stimuli increases for a participant, the standard deviations of his estimates of these stimuli will also increase. This means that if the motoric responses in the present experiment were driven by a ventral stream representation then the standard deviations of the responses of the bars should increase as the lengths are increased. Figure 5, depicting the case for Group 1, show that this does not seem to be the case.

### Is feedback from motoric responses affecting symbolic responses in the double response experiment?

The most parsimonious interpretation of the results of the double response experiment is that a common representation drives both responses. Nevertheless, one alternative interpretation is that separate representations drive each response type but that visual information from seeing the hand making the motoric response (or feedback from the locomotor system when making a motoric response) is somehow influencing the symbolic response in a way that drives the probability of making the same symbolic and motoric response well-above chance. Before we consider this alternative interpretation, it is important to point out that the participant would not know if using the feedback from seeing or feeling the hand making the motoric response would increase the probability of getting the symbolic response right. Therefore, even if possible, there would be no incentive to use the feedback information.

**Figure 6:**
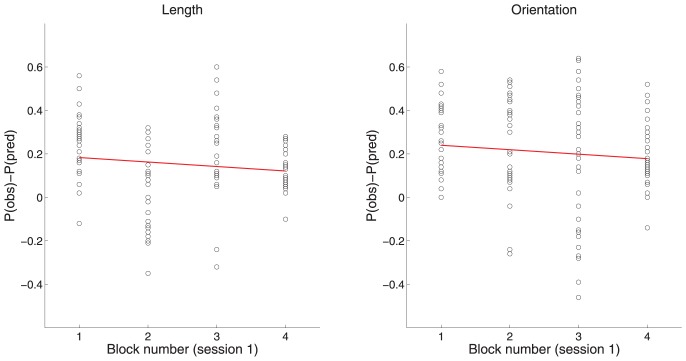
The analysis portrayed in Figure 6 was made because it was conceivable that the well-above chance probability (reported in Figure 3) was driven by transfer of visual information, from seeing the hand making the motoric response, to the systems deciding which symbolic response to make. For such a transfer to occur, though, an association must be formed between visual information, from seeing the hand making the motoric response and a symbolic response. Figure 6 shows that, during the double response trials, no associations are formed between visual information, from seeing the hand making a motoric response and the corresponding symbolic response. If such associations were formed then the associations should become stronger and more precise over time as part of a learning process. This means that the well-above chance probability of making the same symbolic and motoric response should be increasing over time as well as one moves from block 1–4 during part 5 of the experimental procedure where the double responses are made. As can be readily observed, from visual inspection of the simple linear regression fitted to the data (red line), the difference between the observed probability (P(obs)) and the predicted probability (P(pred)) is not increasing as one moves from block 1 to block 4. The data-points were created by reanalyzing the data reported in Figure 3 (for Group 1). Only data from session 1 is part of the analysis (160 trials pr. participant). The 160 trials were presented in four blocks. Within blocks all combinations of length and orientation were shown. For length, each of the data-point consists of 10 trials (160 trials/4 blocks/4 lengths) and for orientation each data-point consists of 8 trials (160 trials/4 blocks/5 orientations). For Group 1 the slope of the red line was negative for both length (−0.025) and orientation (−0.0214). For Group 1, a one-way ANOVA found no significant effect of blocks for either length or orientation (Length: F(3,108) = 1.44, p = 0.236; Orientation: F(3,136) = 1.93, p = 0.127). For Group 2 the slope of the red line was negative for length (−0.057) but slightly positive for orientation (0.0089). A one-way ANOVA did find a significant effect of blocks on the negative slope for length (F(3,108) = 2.89, p = 0.0387) but no significant effect of blocks on the slightly positive slope for orientation (F(3,136) = 2.32, p = 0.0777). Thus, feedback, from the hand making the motoric response, was not driving the well-above chance occurrence of identical motoric and symbolic responses reported in Figure 3.

In order to evaluate quantitatively the likelihood of the alternative interpretation, it is necessary to consider exactly how visual information of the motoric responses could have influenced the symbolic responses so that the probability of making the same symbolic and motoric response was driven well-above chance. First of all, we assume that if the visual information of the hand making the motoric response is to determine a symbolic response, it is necessary to learn to form an association between the visual information of the hand making the motoric response and a symbolic response. After all, it was necessary to help participants, during the training phase, to learn to form associations between the visual information from the presentation of the bar and the symbolic response. In designing the experimental procedure for the training phase, we have tried to make it as hard as possible to learn to form an association between the visual information of the hand making the motoric response and the symbolic response by choosing that no motoric responses were to be made in the trials of the training phase where participants practiced making the symbolic responses. The only motoric responses made during the training phase were those performed when adjusting the diodes for proper registration by the Optotrak sensors and when calibrating the grasps (see procedure). During these motoric responses the correct symbolic responses were not revealed. Nevertheless, during the double response trials (performed after the training phase), the participants might have been able to learn to *appropriately associate* the visual information of the motoric responses with a symbolic response. An *appropriate association* would be one able to influence the symbolic responses to belong to the same category as the motoric response would turn out to belong to, when later analyzed with the Naïve Bayes classifier. The keyword here is learning. Learning to *appropriately associate* is a process that develops in strength and precision over time. So, over time, the participants should become better and better at *appropriately associating* the visual information from the hand making the motoric response with a symbolic response. Consequently, as the learning increases in strength and precision, the probability of making the same symbolic and motoric response should also be increasing. So the question is: did this probability increase? Part 5 of the experimental procedure consisted of four blocks and within blocks all 20 possible combinations of length and orientation were shown once to both sides of the fixation mark (in random order). If participants, over time, become better and better at *appropriately associating* the visual information from the hand making the motoric response with the correct symbolic response, then the difference between the *probability-same-observed* and the *probability-same-by-chance* should increase systematically from block 1 to block 4. In Figure 6 it is clearly shown that no such increase takes place (for Group 1). In fact, for both length and orientation, the slope is the opposite of what should be expected. Thus, in part 5 of the experimental procedure, the alignment of motoric and symbolic responses is not increasing over time. Consequently, the well-above chance probability of making the same symbolic and motoric response (Figure 3) is not observed because the participants transfer visual information from seeing the hand to the systems deciding which symbolic response to make.

## Discussion

### Experimental findings

When presenting a bar, the sensory information projected into the visual system does not in itself represent the length and orientation of the bar. Length and orientation is something that has to be computed. According to the strong segregation version of the perception/action model the visual information (i.e. information before computing representations of length and orientation) reach segregated processing modules underlying symbolic and motoric responses. This means that length and orientation has to be computed independently within each module. If so then the agreement between computations in each of these segregated processing streams should be at chance. Therefore, the present finding (Figure 3) of a highly significant well-above chance agreement for both length and orientation goes against the strong segregation version. The weak segregation version can easily accommodate the findings reported in Figure 3 and as such indicates that a common representation drives both response types.

Given that a common representation drives both response types then why, one might ask, are the responses not in full agreement on all trials? They need not be in full agreement because other factors than the common representation can determine a response. Errors when transforming the common representation into a given response type is one such factor. Also, both types of responses are partly under participants control and can be affected by biases this way. Unconscious biases not under the participants control could also affect the responses and drive them apart.

As can be seen in Table 1 the proportion correct motoric and symbolic responses were very similar. This is further supported by the more detailed analysis comparing (separately for Group 1 and Group 2) the proportion correct motoric and symbolic responses for each category of either length or orientation. This more detailed analysis revealed that only 1 out of 18 paired t-tests showed a significant difference between the two response types. This indicates that the classifications of motoric responses were accurate and validates the method used to gauge length and orientation from motoric responses. Furthermore, it corresponds well with the idea that a common representation drives both response types.

When looking at the responses to length and orientation, for each response type separately, we found clear evidence for independent processing of these features. In fact, the observed probability of getting both length and orientation correct, when making either symbolic or motoric responses, is well predicted by the chance probability of getting both length and orientation correct. That length and orientation are processed independently within both response types is not a direct proof of a common representation since the independent processing could arise independently in two segregated streams (see: *the redundant illusion hypothesis* [27]). Nevertheless, it corresponds well with the idea of a common representation. Furthermore, since the question of whether or not features are reported independently during a motoric responses, to our knowledge, has never before been raised, the finding of independence in motoric responses is in itself an important step forward in the understanding of visual representations driving stimulus directed actions. Apparently, even though grasping an object is perceived as a single unified action (try introspecting on how it feels to grasp something yourself) the necessary computations driving the motoric response to length and orientation are made independently.

According to the perception-action model a ventral stream in the brain drives symbolic responses and a dorsal stream drives motoric responses. Proponents of the perception-action model argue that the dorsal stream is only properly activated by real 3D objects that are visible during most of the motoric response. According to these arguments the brief exposure durations and 2D stimuli used in the double response experiment might have failed to activate the dorsal stream. The proponents of the perception-action model have also argued that the standard deviation of motoric responses to length, as length is increased, does not increase. This is a violation of Weber's law according to which the standard deviations of symbolic responses to length increase as length is increased. Because of this, the proponents of the perception-action model suggest that when standard deviations of motoric responses violate Weber's law it should be seen as a marker for dorsal stream processing. In Figure 5 we show that motoric response to length violate Weber's law. Thus, dorsal stream processing probably drove the motoric responses in the double response experiment.

When contemplating possible confounds in the double response experiment one confound readily presents itself: perhaps the symbolic responses were not always based on the visual impression of the bar. Perhaps instead, sometimes, the symbolic responses were based on the visual impression of seeing the hand making the motoric response to the bar. After all, participants did see their hand making the motoric response immediately before making the symbolic response. Maybe, somehow, this affected the symbolic response? If so, this could have aligned the two response types thus causing the well-above chance finding. For this to occur, though, the participants would have had to learn to associate the visual impression of the motoric response with appropriate symbolic response categories and this would have had to be a learning process. Since learning processes generally increase in strength and precision over time the size of the well-above chance finding should also increase over time. In Figure 6 we show that the well-above chance finding does not increase over time. Consequently, the result in Figure 6 refutes the idea of visual impressions of motoric responses driving symbolic responses. The result in Figure 6, though, is in perfect agreement with the hypothesis of a common representation of length and orientation driving the well-above chance finding.

We note that the present work does not allow for a precise neural definition of what is meant by “common representation”. Schenk, Franz and Bruno [12] suggest a model, opposed to the separate representation model, where visual information is shared across pathways” (i.e. dorsal and ventral red.). Conceptually though, it does not make sense to differentiate between such a model, where different representations in two streams affect each other, and a model where one common set of brain areas, supporting the representation, drive both response types. Assuming that representations, in general, are distributed over processing networks (either large or small) both models portray a single distributed representation shared by both response types.

### Relation to the perception-action model by Milner and Goodale

Many investigations of the coupling between symbolic and motoric responses have related their experimental results to a perception-action model developed by Milner and Goodale [1], [4], [5] here called the perception-action model. We will also follow this trend, but in doing so point out that there are (at least) two versions of the perception-action model. In one version (here called *the strong segregation version*) the representations of length and orientation driving motoric responses are computed in a dorsal stream isolated from representations of length and orientation driving symbolic responses that are computed in a ventral stream [1]–[3], [6], [28], [29]. In another version (here called *the weak segregation version*) the representations of length and orientation driving motoric and symbolic responses are computed before the streams segregate. In the *weak segregation version*, a common representation of length and orientation is therefore fed to the two streams [4]–[8].

In the perception-action model, representations of spatial object features are processed in both streams. This property of the model clearly distinguishes it from earlier dual stream models such as the Ungerleider and Mishkin model where location, a spatial feature of an object, is processed in a dorsal stream and the identity of the object is processed in a ventral stream [1], [9]. Even so, usually, the authors of the perception-action model do not specify whether or not representations of spatial features driving motoric and symbolic responses (such as size, orientation or location) are formed before or after the streams segregate. It only states that the two streams share visual information from V1+. But whether or not the visual information from V1+ contains a representation of a given feature of an object is often underdetermined. Goodale and Milner write: “the two streams share common inputs from early retinotopic cortical areas (orientation, location, size, etc), so that processing of these visual features is not the absolute province of one or other stream” (Goodale & Milner, 2010, pp. 65–66, commentary in [6]). In the above quote it is clear that, in full agreement with *the weak segregation version* of the perception-action model, size is computed before the segregation. But the authors are not consistent on this point. In agreement with *the strong segregation version* of the perception-action model, *size* might also be computed after the streams have segregated: “Although visually-guided grasping of 3D objects requires processing of object size, that computation appears to rely on different neural mechanisms than those involved in the perceptual discrimination of size” [2] (pp12). According to the newest formulation of *the strong segregation version* of the perception-action model [1], it is suggested that visual information can lead to a full analysis of features of an object in the ventral stream without any knowledge of the derived features being transferred to the dorsal stream. Instead the part of the array of visual information where the object is located is flagged. The flagged part of the array of visual information is then analyzed in the dorsal stream (converted into features). A flagged part of the array of visual information does not contain any information on the features of an object. Length and orientation would have to be computed from scratch in the dorsal stream. Based on the results presented in Figure 3, this formulation, of *the strong segregation version* of the perception-action model is implausible.

Because authors often do *not* specify whether they mean the strong or the weak segregation version of the perception-action model the model can explain most results from experiments where participants perform motoric and symbolic responses to stimuli where size or orientation are transformed due to a visual illusion. According to Dyde and Milner [4], [5], [13], “any illusion operating primarily at the early cortical level should inevitably influence feedforward processing throughout the two visual streams (and beyond). Such an illusion should yield associated illusions in both perceptual and visuomotor tasks.” [4] (p. 526) (see also: [29]). Milner and Dyde [5] suggest that researchers, before having participants perform motoric and symbolic responses to stimuli where the experience of size or orientation has been transformed due to a visual illusion, should ask themselves where the likely locus of the illusion is going to be within the brain. But for most if not all illusions we do not know exactly where in the visual system they operate (see [27] for a discussion of the location in the visual system of the Ebbinghaus-illusion). The consequence of the suggestions of Dyde and Milner is that the perception-action model is consistent with illusions both influencing and not influencing action. On the one hand, every time experimental results suggest that motoric responses are fooled by illusions the perception-action model is unchallenged because it can be argued that the illusion is influencing a given spatial feature *before* the streams segregate. On the other hand, when only symbolic responses are affected by the illusion, the perception-action model is supported because it can be argued that the illusion is affecting representations driving symbolic responses *after* the streams have separated.

A consequence of this feature of the perception-action model is that it cannot predict whether or not motoric and symbolic responses to length and orientation, in the double response experiment, should or should not be in agreement well above chance.

Nevertheless, the common representation theory has been pitted against the perception-action model [9], [14], [30], but from the above analysis it follows that they are in fact not necessarily opposed to each other. The perception-action model can take a form (*the weak segregation version* of the perception-action model) where one common neural area, wherein features are both computed and represented, drives both symbolic and motoric responses. According to this version the visual signals separate only for the purpose of *transforming* the common representation into a format suitable for making either a motoric or a symbolic response.

### A note on the use of brief exposure durations

If one wishes to explain the results from the present experiment using *the strong segregation version* of the perception-action model, according to which the parameters of the features are computed separately in two processing streams, an alternative account of the experimental results is necessary.

One possible alternative explanation is perhaps given by the “real-time view of action” hypothesis. According to the originators of this hypothesis (proposed in support of *the strong segregation version* of the perception-action model by [31]): “If the target is not visible when the action is required, the motor control system accesses a stored perceptual representation of the target object that presumably was initially processed by form perception mechanisms in the ventral stream” [13] (pp. 809). The motor control system accesses the ventral stream representation because, according to the “real-time view of action” hypothesis, the dorsal vision-for-action system only computes the exact parametric values of the movement at the very moment the movement is initiated.

In the current experiment the stimulus was presented for 47 ms and the participant moved his hand towards the screen immediately upon stimulus presentation. If these parameters of the experimental paradigm forced the motoric responses to be driven by a stored representation of the bar driving symbolic responses it would explain the above chance alignment of responses since they now both would be driven by a ventrally situated representation.

A movement towards a target, initiated when the target is presented, can be divided into a planning phase and a movement phase. The planning phase starts upon presentation and ends when the hand starts moving. The movement phase starts when the hand starts moving and ends when the target is reached.

Translated into these terms then, according to the “real-time view of action” hypothesis, if the target is visible during the planning phase but not during movement time the dorsal stream still computes the spatial parameters of the target. However, if the target is removed before the planning phase begins, the ventral stream computes the spatial parameters of the target instead of the dorsal stream.

It is not clear the extent to which the bar in the present paradigm can be said to be visible in the planning phase. Nevertheless, it is present during 47 ms of planning time and is unmasked so some sort of cortical iconic image [32] may be part of the planning phase. This means that it is most likely available for some unknown fraction of the planning phase.

At present the “real-time view of action” hypothesis is not sufficiently elaborated to enable a decision as to whether or not, according to the hypothesis, the bar, in the present experiment, was available during enough of the planning phase to engage the dorsal vision-for-action system. Therefore, it is hard to decide whether or not it poses a critique. Nevertheless one can discuss the validity of the critique itself. The main evidence in favor of the “real-time view of action” hypothesis is from a study by Westwood and Goodale [31]. They cued participants to grasp a target among objects in a size-contrast illusion display. When the target was visible in the planning phase (but not during the movement phase) the peak grip aperture during grasping was unaffected by the illusion, but when occlusion of the target coincided with the cue to begin grasping (and the target therefore was not visible during the planning phase), the grasp was sensitive to the illusion. They concluded that dorsal real-time visuomotor mechanisms are engaged only after the planning phase is over, and only if the target is visible during the movement phase. They further argued that when these demands are not met the dorsal action system cannot compute the absolute metrics of the target object and can therefore not resist size-contrast illusions. Instead a ventrally located representation is used for action planning. Because this representation makes use of relational metrics it is sensitive to size-contrast illusions.

Importantly though, Franz and co-workers [33] could not replicate the finding. Instead they found a similar illusion effect in both conditions (target visible during planning phase only or target not visible during either planning nor movement phase). Furthermore, they made the reasonable argument that when the target is visible, during the movement phase, online feedback mechanisms could have registered a possible grasping error created by the illusion and this could have lead to online corrections of the grasp thereby masking an illusion present in this condition as well. In fact, by systematically decreasing visual information of the target, only during the movement phase, from full availability to zero availability, Franz and co-workers could show that the illusion effect increased significantly. The less visual availability the less on-line corrections of the error introduced by the illusion.

To conclude, the use of brief exposure durations does not seriously challenge the assumption, made in the present experiment, that the motoric responses made by participants engage the visuomotor system normally used for grasping objects in the real world.

### A note on the use of 2D stimuli

It has been suggested that one should be cautious when interpreting results of experiments comparing perception and action in which participants grasp 2D stimuli. The rationale behind this suggestion is that the use of 2D stimuli might activate a different set of visuomotor mechanisms than those engaged when using 3D stimuli [34]. While awaiting empirical proof for this suggestion we wish to draw attention to the work of Schenk [35] who discloses a serious problem arising when using 3D objects in experiments directly comparing grasping performance to a symbolic response.

A cornerstone in the evidence supporting the perception-action model is the work done with patient DF [1], [36]. DF has a damaged ventral visual stream and suffers from visual form agnosia. She is unable to correctly report the size of a 3D object, using a symbolic response, but can match the opening of her hand to the size of the object during grasping (motoric response). Because of this single dissociation it was concluded that the neural substrate underlying the use of size in the control of manual skills are distinct from those underlying visual perception of size. In the sentence above we have carefully adhered to the terminology of the original paper reporting the grasping behavior of patient DF [36] to show an example of a the weak segregation version of the perception-action model where size is *used* by the neural substrate underlying a motoric response. As noted earlier, this weak segregation version of the perception-action model is not in disagreement with our finding that, on a trial-by-trial basis, symbolic and motoric responses to length and orientation of a bar are in agreement significantly above chance level. Nevertheless, the point being made here is that the use of 3D objects in experiments directly comparing grasping performance to symbolic responses pose a real problem that does not have to await empirical proof to be substantiated. Schenk [35] demonstrates that the difference found between DF's ability to grasp a 3D block (normal performance) and giving a symbolic response to width (sub-normal) is an artifact of haptic information being available in the grasping task. Removing haptic information equates her performance in the two tasks so that both grasping and symbolic responses become sub-normal [35]. That haptic information is utilized when grasping 3D objects is easily demonstrated: try grasping something with your eyes closed. Choose something that does not move when you touch it. The first time you will be way off target. Next time you will do better. By the third or fourth time grasping will be nearly perfect, solely guided by haptic feedback. The grave problem of haptic feedback from 3D objects points to a great advantage of using 2D stimuli when comparing the task of making a symbolic response to the task of making a motoric response: the tasks are comparable.

## Conclusion

Length and orientation of an object can be specified using two different response types: either by intentional use of symbols or motorically by directly acting upon the object. Here we demonstrate that, on a trial-by-trial basis, symbolic and motoric responses to length and orientation of a bar are in agreement significantly above chance level. This finding is best explained by assuming that symbolic and motoric responses to length and orientation are driven by a common representation. Furthermore, the accuracy of symbolic and motoric responses was very similar for both length and orientation thus corroborating the conclusion of a common representation driving both response types.

Given that a common representation drives both response types, one will expect similar characteristics of the representation to be reflected in both types of responses. One such is the finding that both response types are driven by representations with the same decay characteristics [20]. Our finding that both response types are driven by a representation of objects where length and orientation are processed independently is another shared basic characteristic.

The stimuli used were briefly (47 ms) presented 2D bars. For some this might raise the concern that the neural system driving the motoric responses was not the dorsal stream system normally activated when grasping 3D objects that are visible until the grasp is completed. Instead, perhaps a ventral stream system was driving the motoric responses. If so the well-above chance agreement could be explained as a consequence of a common representation of length and orientation residing in the ventral stream (for both response types). Symbolic response to length follows Weber's law [26], but grasp precision of 3D objects does not [23]. Therefore it has been suggested that motoric responses violating Weber's law can be seen as a marker for dorsal stream processing [25]. The motoric responses to length in the present experiment could not be shown to follow Weber's law (see Figure 5). Therefore, in the present experiment it is most likely that the dorsal stream controlled the motoric responses, indicating that a common representation of length and orientation was driving processing in *both* the dorsal and the ventral stream.

It is conceivable that the well-above chance finding reported in Figure 3 was driven by feedback from the motoric response to the symbolic response. For this to happen, though, the visual impressions of the hand making the motoric response must have been appropriately associated with a symbolic response. Appropriately associating the motoric responses would have had to be a learning process and in a learning process one improves over time. Such an improvement over time should show up in an analysis of the initial double response trials in session one. The well-above chance finding should increase as participants became increasingly better at associating the visual impressions of the hand making the motoric response with the corresponding symbolic response. From Figure 6 it is clear that the well-above chance finding did not increase over time. Consequently, the analysis in Figure 6 refutes the idea that the well-above chance finding could have been driven by feedback from the motoric responses to the symbolic responses.

The question of whether a motoric response and a symbolic response to the spatial features of an object are driven by one common representation or two separate representations is a question directed at the degree of segregation between the neural processing underpinning motoric responses and symbolic responses. The most influential theory, concerning the question of the degree of segregation between the neural processing underpinning motoric responses and symbolic responses, is the perception-action model, by Milner and Goodale [1]. In the section “Relation to the perception-action model by Milner and Goodale” we show that there are in fact two versions of the perception-action model: a version with strong segregation and a version with weak segregation. In the strong segregation version two separately computed representations of spatial features of objects are driving motoric responses and symbolic responses [1]–[3]. In the weak segregation version, a common representation of spatial features of objects, drives both response types [4]–[6]. According to this version the processing of the derived spatial features of objects separates into segregated processing streams only for *transforming* the representations into a neural format suitable for making either a motoric or a symbolic response. Our finding supports the weak segregations claim according to which a common representation drives symbolic and motoric responses to both length and orientation and goes against the strong segregation version of the perception-action model.
